# Feasibility and Safety of Allied Health Professional Delivered Clinics for Suspected Head and Neck Cancers: A Scoping Review

**DOI:** 10.1002/lio2.70362

**Published:** 2026-02-19

**Authors:** Neginsadat Mirtorabi, Dimitrios Spinos, Christopher Coulson, Paul Nankivell, Jameel Muzaffar

**Affiliations:** ^1^ University Hospitals Birmingham NHS Foundation Trust Birmingham UK; ^2^ University of Birmingham Birmingham UK

**Keywords:** 2 week wait, allied health care professionals, FNE, head and neck cancers, remote review, video review

## Abstract

**Background:**

In the UK alone, suspected head & neck cancer referrals exceed a quarter‐million, with a detected malignancy rate of around 2.6%^1^. This, coupled with the need for urgent assessment and treatment of cancer patients, puts an increasing strain on clinical services. Advances in technology have allowed integration of video‐endoscopy, particularly ‘store and forward’ of clinical media and patient information for deferred consultant review. Building on these, Allied Health Professional (AHP)‐delivered nasoendoscopy clinics emerged: AHPs perform endoscopy and a consultant reviews the video asynchronously. This model, in theory, preserves specialist input while helping to address current clinical pressures.

**Methods:**

A scoping review was conducted per PRISMA‐ScR guidelines (PROSPERO 1016067). We searched MEDLINE, EMBASE, PubMed, Emcare, and CINAHL for relevant English‐language studies up to April 2025. Data on clinic setup, diagnostic outcomes, and stakeholder satisfaction were synthesized.

**Results:**

Five papers were included in the study. The majority (*n* = 3) focused on the use of Speech and Language therapists. Across four studies, patients were risk stratified into low and high‐risk groups, with low‐risk patients seen in AHP‐delivered clinics. Three studies focused on cancer diagnostic yield and review within a short time frame. Cancer detection in low‐risk cohorts was minimal, with no missed malignancies. Remote pathways achieved prompt diagnoses (mean 21 days), and the majority of recordings were diagnostically adequate. One study focused on the opinions of ENT surgeons, which was positive, reporting improved efficiency and increased focus on complex cases, but stressed the need for AHP training and robust governance.

**Conclusion:**

Remote asynchronous nasoendoscopy clinics can help meet diagnostic targets and reduce consultant workload. Although robust governance is essential for safe implementation, this model remains a promising and acceptable option. Further work will fully ascertain their long‐term impact on service delivery and patient outcomes.

## 1 Introduction

Between 2022 and 2023, 275,354 suspected Head and Neck (H&N) cancer referrals were made in the United Kingdom (UK), with only 2.6% resulting in a cancer diagnosis [1]. The Faster Diagnosis Standard (FDS) for H&N cancer specifies that imaging and multidisciplinary discussion should be completed within 28 days of referral [2]. Given the relatively low cancer conversion rate, the associated anxiety for patients, and the considerable clinical and administrative workload of assessment, there is a clear need for redesigned pathways that deliver safe, streamlined care.

The COVID‐19 pandemic accelerated the development of alternative referral models, including telephone triage [3], risk‐stratification calculators [4], and one‐stop neck lump clinics [5]. Technological advances also enabled wider use of video‐endoscopy, particularly store‐and‐forward systems for uploading clinical media for delayed consultant review [6]. Building on these innovations, increasing attention has been directed toward expanding the role of Allied Health Care Professionals (AHPs) in initial assessment pathways. Under this model, AHPs conduct the clinical examination and consultants subsequently review the recorded images and videos asynchronously. With only 2.19 otolaryngology–head and neck consultants per 100,000 population globally [7], consultant time is an increasingly scarce resource. Asynchronous review models preserve specialist oversight while reducing pressure on consultant‐led clinics. Comparable approaches have been successfully piloted in other ENT subspecialties, including otology and rhinology [6].

Introducing such pathways requires careful planning to safeguard clinical standards. Logistical considerations—including triage criteria, digital image handling and transfer, long‐term follow‐up arrangements, and comprehensive AHP training—must be addressed to ensure quality and safety. This scoping review evaluates the feasibility and diagnostic safety of AHP‐delivered clinics with asynchronous consultant review. It further examines the range of remote formats trialed, their adherence to clinical governance standards, and their acceptability to clinicians and patients.

## 2 Material and Methods

A scoping review was conducted and reported based on the PRISMA‐ScR checklist. This scoping review is registered on PROSPERO with registration number 1016067.

A literature search was conducted across Medline, EMBASE, Emcare, PubMed, and CINAHL. Search terms included, but were not limited to: “FNE,” “2‐week wait,” “head and neck cancers,” “allied health care professionals,” “remote review” and “video review” (Appendix A). These terms were used in various combinations to ensure the retrieval of relevant studies across all selected databases. The review focused on studies published in English, within the past 25 years, with an end‐date on April 2025.

All retrieved records that met the inclusion criteria were independently screened for relevance and quality by two reviewers. Any discrepancies were resolved through discussion, facilitated by a third reviewer. Data extraction focused on study characteristics, participant demographics, methodology, and outcomes. The primary outcomes of interest were risk stratification, stakeholder satisfaction, and clinical feasibility and safety. The heterogeneous nature of included papers with respect to methods and outcomes meant analysis was restricted to qualitative synthesis of themes, following principles of grounded theory.

## 3 Results

**FIGURE 1:**
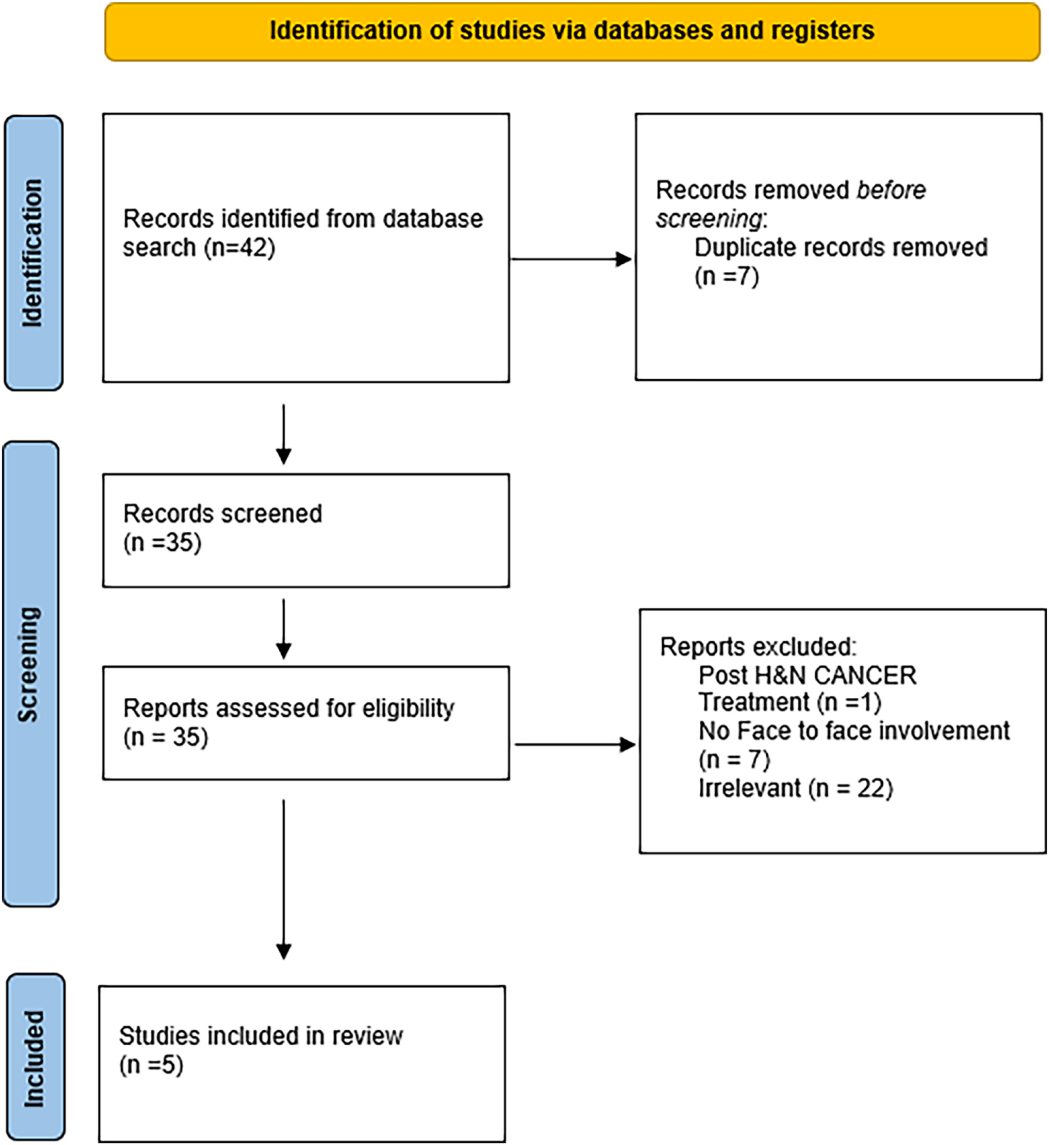
PRISMA flow chart.

Forty‐two papers were identified, with seven duplicate results. Two results were returned through EMBASE, 19 through Ovid MEDLINE, and 21 through PubMed (Figure 1). Exclusion criteria included any paper that did not specifically relate to H&N cancer pathology, did not include remote assessment by clinicians, and those which did not include use of video imaging. To ensure review of the most relevant studies, inclusion criteria consisted of any papers published between 2000 and 2025, in English, involving H&N cancer adult patients. Based on these criteria, five papers were included in the study.

Five papers were included in the study, with the publication date ranging from 2019 to 2025 (Table 1). All papers were published within the UK. Overall, three studies [8, 9, 10] focused on use of Speech and Language Therapists (SLTs) while the other two [11, 12] looked at use of nurses in H&N cancer clinics. One paper [10] focused on the scope of SLTs in their ability to initiate management for specific red flag symptoms such as hoarseness of voice.

**TABLE 1:** Included studies.

First author	Year	Country	Number of patients/participants	Modality of service	Type of study
Christopher Metcalfe	2022	United Kingdom	661 (total referrals, 340 telescopic)	Novel telescopic pathway vs. conventional standard of care	Prospective service evaluation
Jen Butler	2023	United Kingdom	218	SLT‐delivered 2‐week wait assessment clinic	Prospective pilot clinic
Christopher Metcalfe	2024	United Kingdom	660 (low‐risk patients)	Nurse‐delivered clinic with asynchronous consultant review	Prospective service analysis
Louise C. Occomore‐Kent	2025	United Kingdom	11 ENT surgeons	Exploration of views of ENT surgeons on SLT‐delivered first point of contact clinic	Qualitative study
Louise Occomore‐Kent	2021	United Kingdom	NA	SLT‐delivered 2‐week wait assessment clinic	Opinion on pilot study

Across the reported studies, three used a triage system [9, 12] or the Head and Neck Cancer Risk Calculator (Version 2 (HaNC‐RC v.2)) [11] prior to assigning patients to traditional clinician‐led clinic models or to remote/asynchronous review.

One study [8] specifically assessed stakeholders' satisfaction by looking at the acceptability of AHP‐delivered clinics by ENT consultant surgeons. In this paper, thematic analysis revealed that the surgeons identified potential benefits, including improved governance, early cancer detection, cost savings, reduced workload for ENT surgeons, and the utilization of existing SLT resources [8]. However, concerns were raised regarding the legal authority for SLTs to make independent mucosal diagnoses, variable levels of SLT competence in differentiating benign from concerning symptoms, and patient expectations [8].

In terms of outcome measures, 3 studies [9, 11, 12] focused on the cancer diagnostic yield and review within a short time frame. The diagnostic rate was reported to be less than 1% for 715 patients [11, 12]. Butler et al. [9] had a higher cancer diagnostic yield of 5%, with high‐grade dysplasia detected in 3% of cases. Images and management plans could be reviewed and formulated in almost 1 week [9] and the mean waiting time was demonstrated to align with the NHS cancer pathway targets [2, 11]. Metcalfe et al. further demonstrated a shorter time to diagnosis compared to the standard pathway [11].

Cancer detection rate and re‐referral was low across the reviewed papers. In Metcalfe et al. prospective analysis, six head and neck cancers were diagnosed (0.9% conversion rate) from the 660 low risk patients [12]. Remote videos were deemed adequate in 98.9% [12]. During a mean follow‐up of 15 months, 21 patients (3.2%) were re‐referred; no head and neck cancers were identified on re‐referral, and there were no missed cancers in the follow‐up period [12]. In another study, out of the 155 low risk patients, no cancer was diagnosed (< 1%), with no re‐referrals within three months [4]. Among high‐risk consultant‐led patients, 31 cancers were identified (17% conversion rate) [11]. In the **c**onventional consultant‐delivered standard‐of‐care pathway during the same period, 74 patients were reviewed face‐to‐face, with four cancers detected (5.4%) [11].

All five papers [8, 9, 10, 11, 12] commented on the acceptable safety profile of AHP‐delivered cancer clinics. There was no requirement for a second ENT opinion [9] and no new cancer diagnosis within follow up periods [11, 12].

## 4 Discussion

**FIGURE 2:**
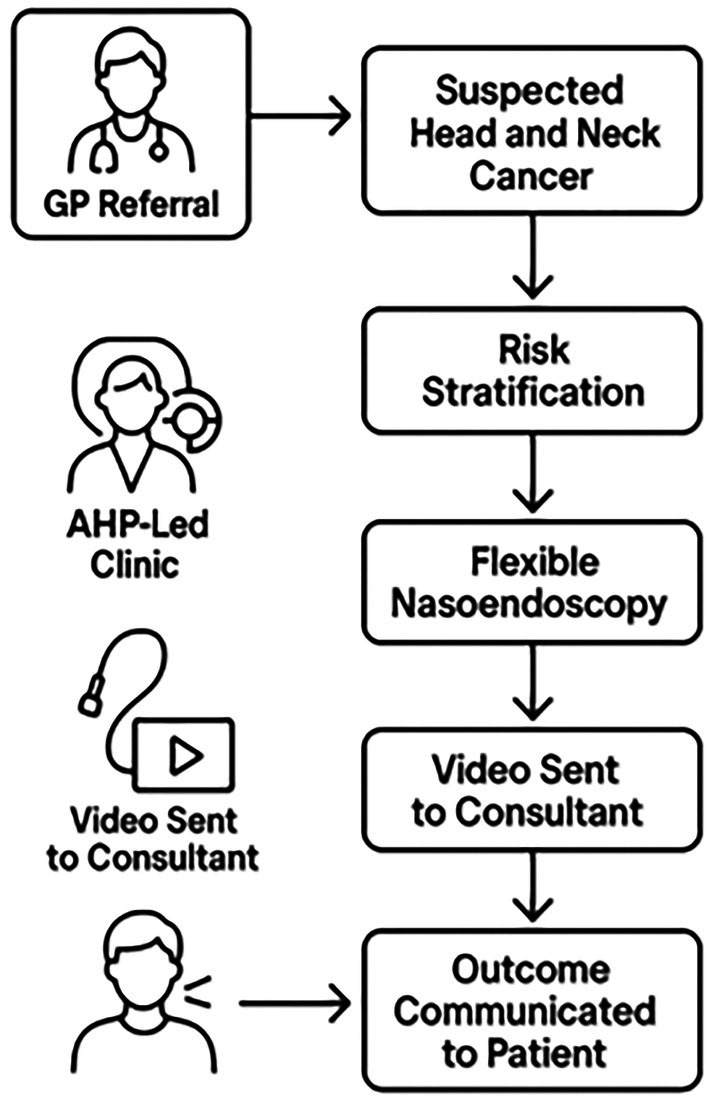
Sample algorithm of AHP‐delivered clinic.

**FIGURE 3:**
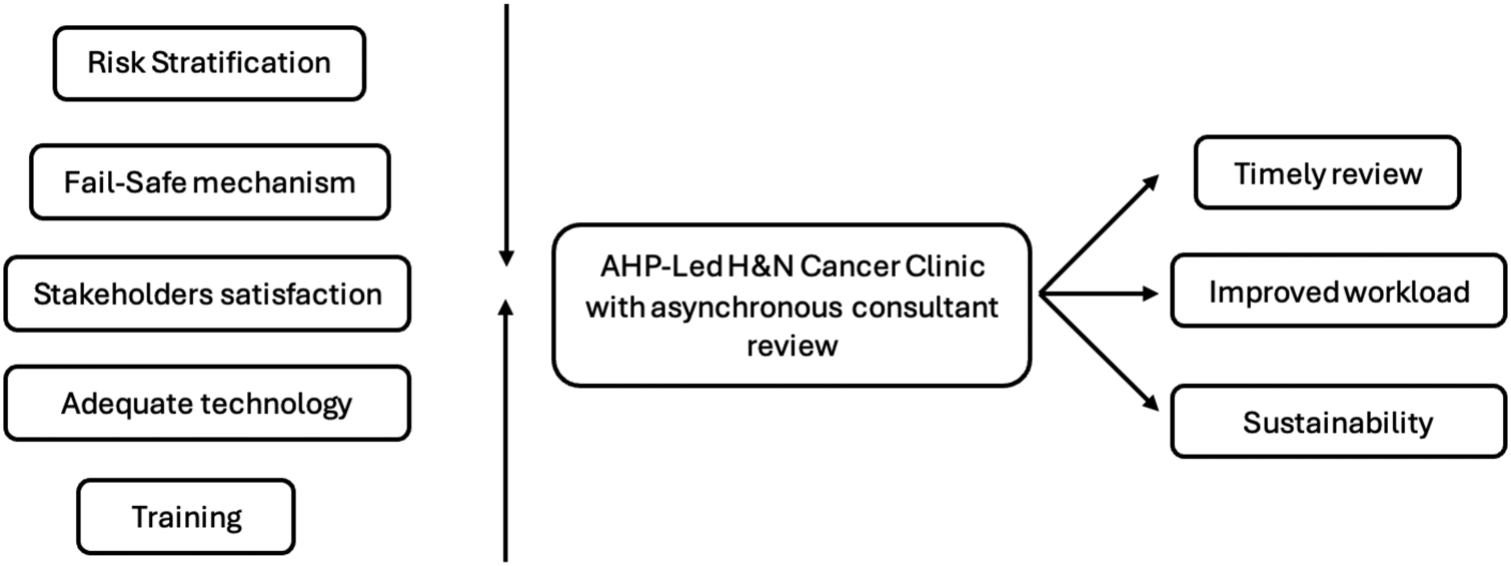
Feasibility of AHP‐delivered H&N clinics and potential benefits.

This scoping review examined the feasibility of remote asynchronous review of nasoendoscopes for suspected H&N cancer patients (Figure 2). Although there have been a limited number of papers published on this subject, the evidence so far suggests this is a safe, effective, and reproducible approach. The importance of risk stratification, stakeholder satisfaction, consistent clinical standards, failsafe strategies, and sustainability need to be addressed (Figure 3).

### 4.1 Clinical Efficacy and Feasibility

#### 4.1.1 Risk Stratification

Effective risk stratification is critical for selecting appropriate patients for remote review clinics. During the COVID‐19 pandemic, the British Association of Head and Neck Oncologists (BAHNO) advocated the use of a validated H&N cancer risk calculator to support patient stratification [4]. One commonly employed approach is symptom‐based risk assessment, which evaluates the presence and severity of specific clinical indicators such as persistent hoarseness, unexplained weight loss, or non‐healing ulcers [13]. In a prospective population‐based study, Hardman et al. demonstrated that remote triage incorporating symptom‐based stratification safely managed suspected H&N cancer referrals, as evidenced by a low conversion rate to cancer diagnoses, thereby reducing unnecessary hospital attendance for low‐risk patients [14]. It is important to highlight that the location of the suspected lesion should play a part in risk stratification. Asynchronous endoscopic review is most appropriate for subsites of the upper aerodigestive tract that are accessible via flexible nasendoscopy, rather than for all potential head and neck cancer presentations. As highlighted in Metcalfe et al. [11, 12] the telescopic pathway was specifically designed for low‐risk referrals requiring visualization of the larynx and pharynx. Patients presenting with neck masses without an obvious primary or lesions confined to the oral cavity or tonsils were triaged as high‐risk and either directed to face‐to‐face consultant assessment or primary imaging prior to review. Similarly, Butler et al. [9] and Occomore‐Kent et al. [8] emphasized that SLT‐led clinics are best suited to evaluating pathologies of the larynx and pharynx, where endoscopic visualization is possible and within established SLT competencies, rather than intra‐oral or submucosal lesions.

Another tool, the Head and Neck Cancer Risk Calculator (Version 2), is a statistical model that predicts an individual's H&N cancer risk by generating a percentage score based on symptoms, demographics, and lifestyle factors [4]. This calculator aids in directing patients to the appropriate referral pathways, either for urgent suspicion of cancer or for routine care. However, Ying Tan et al. reported that integrating this tool into primary care settings may increase the number of urgent referrals, even when the absolute risk remains low [15].

With the growing influence of artificial intelligence (AI), machine learning (ML) models have also been applied to risk stratification both before and after diagnosis. Notably, variational logistic regression predicts an optimal balance between the accurate identification of patients without cancer (high true negative rate) and minimisation of the misclassification of patients with cancer (low false negative rate) [16]. ML models may be applied to complex datasets, containing such data as clinical symptoms, imaging, and patient demographics, to enhance the predictive capacity of probabilistic classification [16]. This refined classification in turn improves diagnostic accuracy, expedites referrals, and optimizes treatment pathways. Improved identification of true negatives creates further opportunity for the development of alternative pathways within secondary care for managing non‐cancerous diagnoses more efficiently [16]. Such improvements could potentially reduce the overall referral rate from primary care, leading to significant efficiency savings [16].

#### 4.1.2 Image Quality

Remote videoendoscopy, whether synchronous or asynchronous, has been trialed in other areas of ENT, including otology and rhinology [7]. Indeed, Miller et al. focused on the feasibility of remote nasendoscopy in pediatric patients [17]. In their study, 45 pediatric patients underwent nasopharyngolaryngoscopy. The examination was recorded and a second remote otolaryngologist reviewed the images [17]. Results showed adequate diagnostic accuracy and smooth operation. Elsewhere, Kokesh et al. found video‐otoscopy images to be comparable to an in‐person examination [18].

Video imaging increases the potential for dynamic assessment pathways, particularly in such cases as vocal cord pathologies. Clearly, image and video quality are pivotal to the viability of such pathways. For a consultant to make an accurate diagnosis remotely, images must be consistent and sufficiently detailed. In the reviewed studies, the quality of videos and images was not assessed formally.

Image data must also be stored in a sufficiently small size to enable transfer, without impacting quality. Metcalfe et al. used video compression to improve storage and data transfer, notably allowing for the storage of videos in patients' records for the optimisation of communication and assessment during multi‐disciplinary team (MDT) meetings and discussion [12].

#### 4.1.3 Safety

An even more important question to be answered when assessing feasibility is whether this method is safe at detecting cancers, and what fail‐safe mechanisms are needed to mitigate risks associated with remote assessments, such as misdiagnosis or missed cancers. Studies by Butler et al. [9] and Metcalfe [12] examined the safety profile of remote assessment. The rate of high‐risk diagnosis in patients triaged to be seen by AHPs was low, but the absence of re‐referrals indicates that the clinic's assessments were comprehensive and accurate [9, 12]. In Metcalfe et al. study, there were no missed cancers during the follow‐up period, underscoring the safety of the proposed pathway [12]. The pathway also met the 28‐day faster diagnosis standard, demonstrating its efficacy in timely cancer detection. The asynchronous review of images within 1 week in the reviewed studies highlights the safety netting available [3].

#### 4.1.4 Training

Training AHPs to safely assess patients and perform FNEs is of paramount importance in ensuring the feasibility of remote clinics. Adequacy of training, professional recognition of AHPs, and the establishment of robust governance structures to ensure patient safety and quality of care are challenges that must be addressed [8]. SLT practitioners already get training to perform fibreoptic endoscopic evaluation of swallowing (FEES) [19], and expanding these skills to the performing of FNE should be eminently practical. The use of simulation when training allows practitioners to gain confidence and repetitive experience in performing endoscopy without compromising patient care [19]. Despite this, there is very limited focus in the literature on the ideal training curriculum and requirements needed to ensure that AHPs are adequately prepared.

### 4.2 Stakeholder Satisfaction

For remote pathways of care to be successful, all stakeholders, including both clinicians and patients, must be satisfied. Studies indicate that clinicians generally accept remote assessment pathways, provided they are well‐structured and integrate seamlessly with existing workflows. Certainly, O Commore‐Kent et al. semi‐structured interviews with 11 UK‐based ENT surgeons highlighted a generally favorable view [8]. Workforce availability and organizational support were vital to the implementation and success of these clinics [8]. On the whole, consultants acknowledged potential advantages, including efficient management of low‐risk patients, reduced waiting times, and the effective use of AHPs' specialized skills in laryngeal examination and flexible nasoendoscopy [8].

The satisfaction of patients with remote review is as critical a consideration as that of clinicians. Although specific data on patient satisfaction were limited in the reviewed studies, convenience and reduced commuting are likely to be well‐received by patients [20, 21]. Smith et al. highlighted early diagnosis and anxiety resolution as particular factors that led to higher patient satisfaction [22]. If patients triaged into low‐risk groups are seen in the remote pathway, a higher proportion of consultants' time will be spent seeing those with more complex needs. This allows more time to be spent on those consenting to potential interventions or an explanation of their diagnosis, whereas patients with benign pathology can be reassured remotely in a timely manner. If a credible technician can procure data that are reviewed by a consultant, and results are communicated to patients by appropriate individuals in a timely manner, patient satisfaction should not be impacted negatively. For such an outcome to be achieved, it must be ensured that patients are adequately informed and comfortable with remote procedures.

The view of AHPs is another important consideration. Bradley et al. in their survey of 129 SLTs from all regions of the UK, found overall enthusiasm for expanding the scope of practice, if there is adequate training and supervision [23].

Another, somewhat less direct aspect of stakeholder satisfaction is the impact of this pathway on surgical training, particularly the training of future head and neck surgeons. While current patients will receive consultant‐led care, whether face‐to‐face or remotely, trainees may see a reduction in the number of H&N cancer referrals they encounter and assess [24]. On the other hand, recordings can provide a valuable learning resource where cases can be discussed and learnt from [25].

### 4.3 Sustainability

Another point in favor of streamlining cancer referral pathways and remote scoping review is the matter of sustainability. NHS England's Green initiative, as detailed in its Delivering a ‘Net Zero’ NHS *strategy* (NHS England, 2021) [26], recommends the transformation of outpatient services through telemedicine and digital platforms to reduce carbon emissions. Gerrard et al. quantified the environmental benefits of changing 100 appointments from face‐to‐face to remote, identifying reductions of 2150 patient travel miles, 47 occupied hospital car parking spaces, and 0.4 *t* of CO_2_ emissions. In this model, care can be delivered outside the hospital and therefore potentially reducing miles traveled. Transitioning to remote healthcare consultation on a larger scale should have proportionately significant ecological advantages [27].

Similarly, the Royal College of Surgeons underscores the importance of environmental sustainability in *its* Sustainability and Environment Report (Royal College of Surgeons, 2022), advocating the integration of remote consultation technologies within surgical pathways [28]. A systematic review by Ravindrane et al. found that all 14 included studies reported environmental benefits to telemedicine over face‐to‐face consultations, primarily through reduced greenhouse gas emissions from travel [29].

### 4.4 Future Perspectives

Despite the limited number of studies identified that focused on the use of AHPs performing FNEs in H&N cancer clinics, the overarching theme of those reviewed here is that low‐risk patients could safely be seen by AHPs, with images reviewed shortly after by a consultant. The low rate of cancer diagnosis in patients identified by remote review as low‐risk further highlights both the efficacy of triage systems, whilst the low rates of re‐referral and missed cancers highlight the proficiency and safety of AHP‐led clinics.

Our review identified three main gaps in the literature. Firstly, there is no formal study on the cost‐effectiveness of this remote review compared to traditional pathways. Although asynchronous review may be cost‐effective at the time that it is performed, the cost of setting up AHP‐delivered clinics, equipment and training must be considered. Secondly, more studies are needed to evaluate the views of AHPs in the expansion of their practice, the training and responsibility that would come with this expansion, and the implications for AHP workforce planning. Finally, there are few adequate validation studies on the software needed for recording, storing and transferring imaging data.

## 5 Conclusion

This scoping review demonstrates that AHP‐delivered nasoendoscopy for suspected H&N cancer patients with asynchronous consultant review is a safe and promising approach. This is, however, contingent on the integration of robust risk stratification, stakeholder engagement, and stringent clinical governance. Such pathways support faster diagnosis and operational efficiency while aligning with broader aspirations to modernize care delivery and reduce environmental impact. Overall, the evidence supports the clinical efficacy and safety of remote pathways, although further research is required to refine these protocols and assess their long‐term impact on service efficiency and patient outcomes.

## Conflicts of Interest

The authors declare no conflicts of interest.

## Data Availability

The data that support the findings of this study are available from the corresponding author upon reasonable request.

## Appendix A Detailed Search Strategy

Search Strategy and notes:

1. Topic:

Use of remote consultant review in head and neck cancer 2 weeks wait clinics whereby an allied healthcare professional does the nasoendoscopy and a consultant will remotely review the videos.

2. Keywords or possible Search Terms

FNE

2 week wait

Head and neck cancers

Allied health care professionals

Remote review

Video review

Search limits

Any paper published in the last 25 years

Sources searched (number of results in brackets):

Embase (2)

Ovid MEDLINE (19)

Pubmed (21)

Date range: 25 years

Limits: None

Ovid MEDLINE(R) ALL <1946 to April 01, 2025>

1 “2 week* wait”.ti,ab. 166

2 “2‐week* wait”.ti,ab. 166

3 “two week* wait”.ti,ab. 131

4 “two‐week* wait”.ti,ab. 1315 “2ww”.ti,ab. 82

6 “2 ww”.ti,ab. 40

7 “2‐ww”.ti,ab. 40

8 “two‐ww”.ti,ab. 63

9 “two ww”.ti,ab. 63

10 “twoww”.ti,ab. 0

11 1 or 2 or 3 or 4 or 5 or 6 or 7 or 8 or 9 or 10 391

12 “remote review”.ti,ab. 63

13 “video review”.ti,ab. 586

14 “video appointment”.ti,ab. 13

15 telehealth.ti,ab. 13837

16 “digital consultation”.ti,ab. 33

17 “digital appointment”.ti,ab. 7

18 “virtual consultation”.ti,ab. 192

19 “virtual appointment”.ti,ab. 33

20 telerehabilitation.ti,ab. 2104

21 telehealth.ti,ab. 13837

22 “video consultation”.ti,ab. 469

23 “video call”.ti,ab. 453

24 videoconferenc*.ti,ab. 4381

25 “video conferenc*”.ti,ab. 2017

26 “remote consult*”.ti,ab. 914

27 “remote assessment”.ti,ab. 572

28 12 or 13 or 14 or 15 or 16 or 17 or 18 or 19 or 20 or 21 or 22 or 23 or 24 or 25 or 26 or 27 23845

29 nasoendoscopy.ti,ab. 240

30 nasendoscopy.ti,ab. 327

31 nasendscopy.ti,ab. 1

32 Nasendoscopic.ti,ab. 70

33 flexible nasal endoscopy.ti,ab. 40

34 “nasal endoscopy”.ti,ab. 2187

35 29 or 30 or 31 or 32 or 33 or 34 2785

36 “head and neck cancer”.ti,ab. 30869

37 “head and neck oncologyv”.ti,ab. 801

38 36 or 37 31386

39 “speech and language therap*”.ti,ab. 2578

40 SLT.ti,ab. 2808

41 AHP.ti,ab. 3880

42 “allied health professional*”.ti,ab. 2909

43 39 or 40 or 41 or 42 11575

44 11 and 28 and 38 2

45 11 and 38 and 43 4

46 11 and 35 and 38 4

47 11 and 35 4

48 11 and 38 35
